# Child Abuse Pediatrics Fellowship Recruitment: Interview Structure and the Use of Multidisciplinary Interviewers

**DOI:** 10.7759/cureus.110120

**Published:** 2026-06-02

**Authors:** Dena M Lowing, Kristin Garton Crichton

**Affiliations:** 1 Pediatric Medicine, University of North Carolina Hospitals, Chapel Hill, USA; 2 Child Abuse Pediatrics, Nationwide Children's Hospital, Columbus, USA

**Keywords:** child abuse pediatrics fellowship, fellowship recruitment, graduate medical education (gme), interview best practices, multidisciplinary interviewers

## Abstract

Introduction: Graduate medical education (GME) programs aim to improve equity, reduce bias, and assess applicants holistically, yet implementation of evidence-based interview practices varies. Multidisciplinary collaboration is foundational to many medical specialties, especially child abuse pediatrics (CAP), but the extent to which multidisciplinary perspectives are incorporated into fellowship interviews is not well described. This study aims to describe fellowship interview structures, the use of multidisciplinary interviewers, and the factors influencing applicant ranking in CAP.

Method: A national cross-sectional survey of all CAP fellowship program directors (PDs) was conducted in 2023 regarding the 2022-2023 interview season. Eligible participants included PDs from all 33 accredited CAP fellowship programs. The survey assessed interview structure, use of standardized practices, interviewer composition, and factors influencing applicant ranking. Descriptive statistics were used to summarize responses.

Results: Seventeen of 33 eligible PDs responded (52%). Interview structures varied, with inconsistent use of standardized questions (10/17, 59%), scoring rubrics (6/17, 35%), blinded interviews (5/17, 29%), and formal interviewer training (8/17, 47%). Most programs (10/17, 59%) incorporated interviewers outside the core physician faculty, most commonly social workers (9/17, 53%), advanced practice providers (7/17, 41%), and current fellows (6/17, 35%). Interview quality and recommendation letters from CAP faculty were among the most influential factors in ranking applicants, whereas publications were the least influential.

Conclusions: Fellowship interview practices demonstrate substantial variability, including inconsistent application of evidence-based methods. The frequent use of multidisciplinary interviewers suggests an emerging recruitment approach aligned with interprofessional care models that may have relevance across GME settings.

## Introduction

Fellowship interviews play a central role in trainee selection across graduate medical education (GME) and are increasingly viewed as an opportunity to support holistic review, equity, and alignment with workforce needs. While structured interview practices have been recommended to improve fairness and reliability, their uptake across training programs remains inconsistent. At the same time, many medical subspecialties emphasize interprofessional collaboration in clinical care, yet interviewer composition during fellowship recruitment has received relatively little attention.

A growing body of evidence supports the use of structured interview practices, such as interviewer training, standardized questions, scoring rubrics, and blinded interviews, which have been shown to reduce bias, increase diversity, and improve recruitment outcomes [1-4]. However, adoption of these practices across GME is inconsistent [5,6]. In parallel, interprofessional collaboration is a core competency in medical training, designated as Entrustable Professional Activity 9 by the Association of American Medical Colleges [7]. Despite this emphasis, little is known about whether multidisciplinary perspectives are incorporated into fellowship interviews. Limited studies outside of GME suggest that multidisciplinary interview panels may reduce individual bias and promote more comprehensive assessment of candidates, but this approach has not been well studied in fellowship recruitment [8,9].

Child abuse pediatrics (CAP) is inherently team-based, relying on close collaboration among physicians, social workers, advanced practice providers, mental health professionals, and other disciplines [10,11]. As such, CAP provides a useful model for examining how multidisciplinary perspectives are incorporated into fellowship interviews. However, there is limited literature describing interview structures, interviewer composition, and ranking practices within CAP fellowship recruitment. To address this gap, this study aims to describe fellowship interview structures, the use of multidisciplinary interviewers, and the factors influencing applicant ranking in CAP.

## Materials and methods

This is a national cross-sectional survey of fellowship program directors (PDs) of all 33 Accreditation Council for Graduate Medical Education-accredited CAP fellowship programs regarding the 2022-2023 interview season. The study was conducted in 2023. CAP was selected as a model subspecialty because of its reliance on interprofessional clinical practice.

The 15-item survey assessed interview structure, interviewer composition, and applicant ranking criteria (administered via REDCap (Research Electronic Data Capture; Vanderbilt University, Nashville, Tennessee, United States); full survey instrument provided in the Appendices). Items addressed use of standardized questions, scoring rubrics, interviewer training, blinded interviews, and participation of multidisciplinary interviewers. Lastly, CAP fellowship PDs were asked to rate nine factors used in ranking applicants on a scale of 1 to 9 (1=most valuable; 9=least valuable), with each value assigned once to create a ranked order. Survey development followed best practices, including expert review and pilot testing with clinician educators familiar with fellowship recruitment [12,13]. The survey was distributed to fellowship PDs via the Ray E. Helfer Society listserv, a national scholarly organization for CAP, with an initial email invitation sent in June 2023, and two reminder emails were sent to encourage participation. Completion required approximately 5-10 minutes, and no incentives were offered. 

Descriptive statistics were used to summarize responses. For PDs who submitted more than one response, only the most recent submission was included. This study was reviewed by the Nationwide Children's Hospital Institutional Review Board (STUDY00000676) and determined to be exempt.

## Results

Of the 33 CAP fellowship PDs surveyed, 17 responded (17/33, 52%), representing programs in 12 geographically diverse states. Respondents reported variability in program size and interview participation. The number of faculty full-time equivalents (FTEs) varied, with most programs reporting between 2.1 and 5.0 FTEs (7/17, 41%). The number of faculty participating in interviews was most commonly five to eight (9/17, 53%). 

Most programs reported interviewing 1-10 applicants annually over the prior three years (15/17, 88%), while two programs interviewed between 11 and 15 applicants (2/17, 12%). Eleven programs enrolled an average of one fellow per year (11/17, 65%), three programs enrolled one fellow every other year (3/17, 18%), two programs enrolled one fellow every three years (2/17, 12%), and one program enrolled two fellows annually (1/17, 6%). 

Interview day structures varied across programs. Most programs conducted half-day interview sessions (14/17, 82%), with one applicant interviewed per day in the majority of programs (15/17, 88%). Applicants typically completed multiple interviews per interview day, most commonly seven (7/17, 41%).

**Figure 1:**
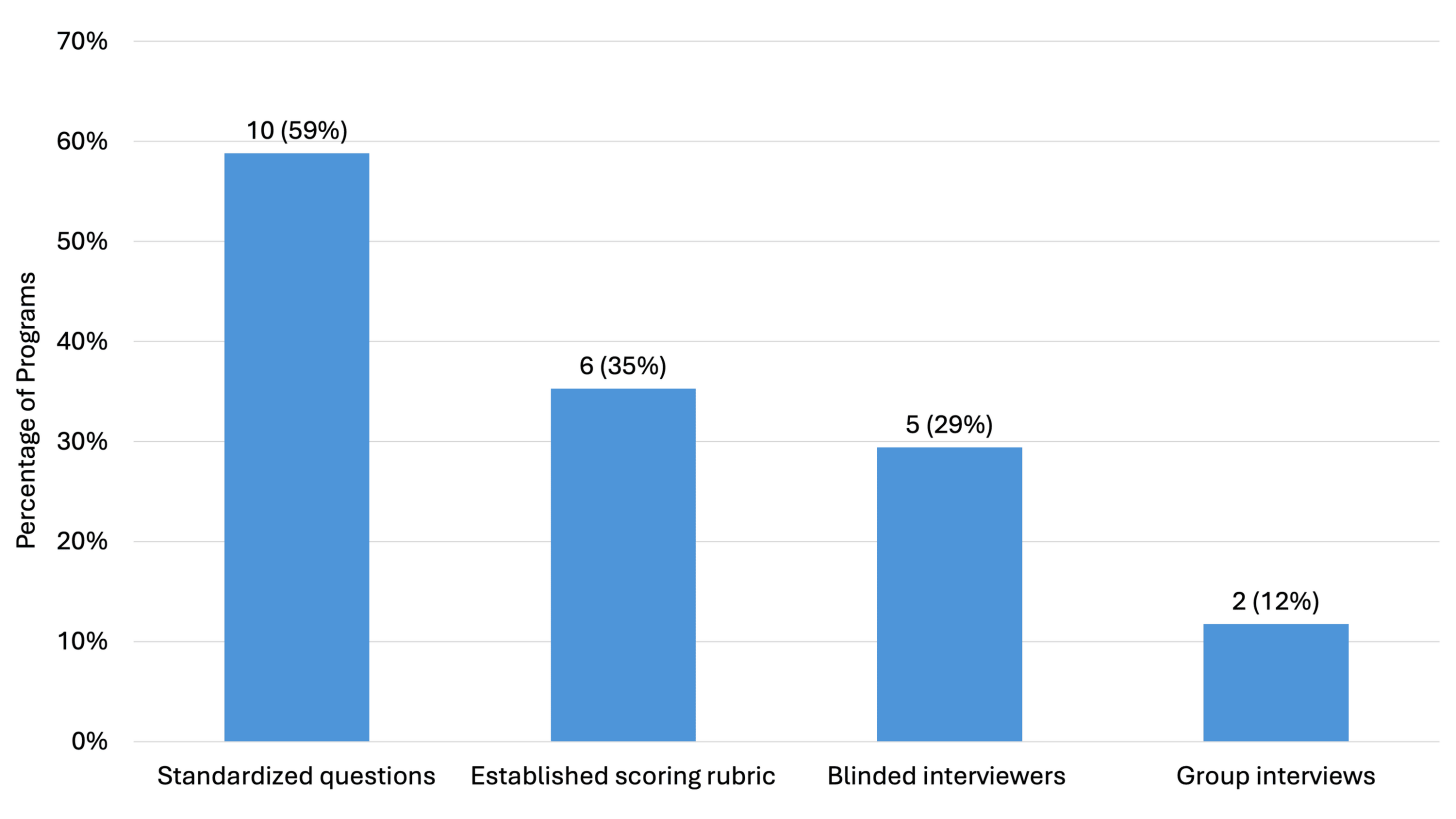
Interview structure of national child abuse pediatrics fellowship programs

Standardized or structured interview questions were used by 10 programs (10/17, 59%) (Figure 1). Six programs used an established scoring rubric (6/17, 35%), and five programs used blinded interviewers, defined as having limited knowledge of some or all aspects of the applicant's application materials (5/17, 29%). Among programs using blinded interviews, interviewers most commonly had restricted access to the MyERAS application, Medical Student Performance Evaluation (MSPE), board transcripts (United States Medical Licensing Examination (USMLE) or Comprehensive Osteopathic Medical Licensing Examination of the United States (COMLEX-USA)), and medical school transcript (each 4/5, 80%), as well as the applicant photo (3/5, 60%). Two programs conducted group interviews in which multiple applicants were interviewed simultaneously (2/17, 12%), and no programs reported using multiple mini-interviews. Formal interview training was reported by eight programs (8/17, 47%), described as one to two training sessions led by the PD, departmental faculty, and/or institutional staff.

**Figure 2:**
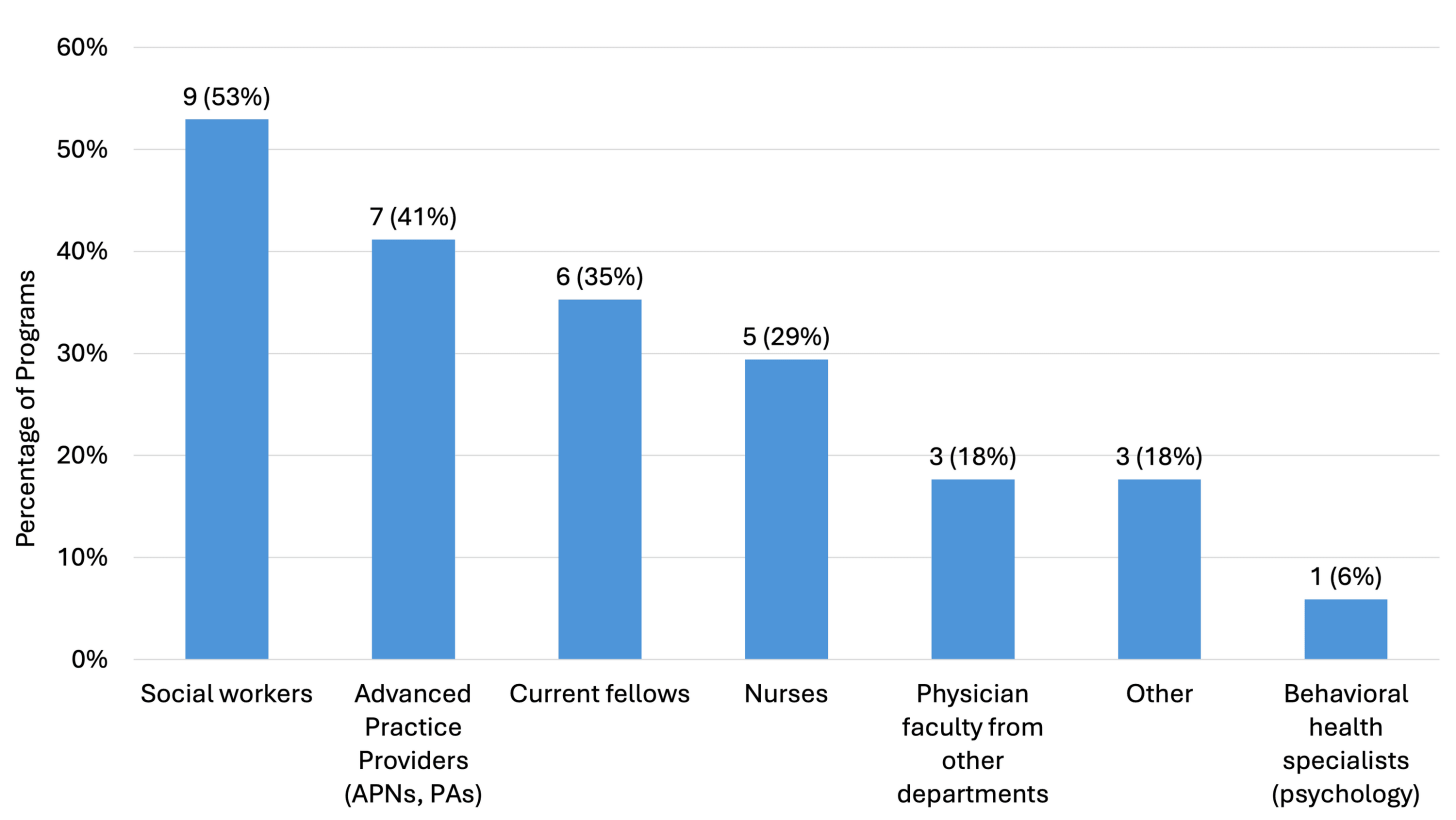
Types of non-departmental faculty interviewers for national child abuse pediatrics fellowship programs APNs: advanced practice nurses; PAs: physician assistants

Most programs incorporated interviewers outside of their core departmental faculty (10/17, 59%). These included social workers (9/17, 53%), advanced practice providers (7/17, 41%), current fellows (6/17, 35%), nurses (5/17, 29%), physician faculty from other departments (3/17, 18%), administrative assistants or program coordinators (2/17, 12%), behavioral health specialists (1/17, 6%), and child life specialists (1/17, 6%) (Figure 2). Among the programs using non-physician interviewers, six conducted panel interviews involving two or more interviewers (6/17, 35%).

Twelve programs completed rankings of applicant selection criteria for creating their rank-order list. The highest-ranked factors were a letter from a CAP attending who had worked with the applicant and interview quality, while publications were ranked least important (Table 1). 

**Table 1:** Selection criteria used to rank child abuse pediatrics fellowship applicants CAP: child abuse pediatrics; USMLE: United States Medical Licensing Examination; COMLEX-USA: Comprehensive Osteopathic Medical Licensing Examination of the United States Selection criteria were ranked on a scale from 1 to 9, with 1 representing the most important factor and 9 the least important. Median rank scores are presented for each criterion. Lower median scores indicate greater relative importance.

Selection criteria	Median rank score
Letter from a CAP attending whom the applicant works with	3
Quality of interview	3.5
Letter from the director of the applicant's residency program	5
Personal statement	5
Quality of residency program	5
Applicant has expressed interest in the program (telephone call, email)	5
USMLE/COMLEX-USA scores	5.5
Medical school rank	6.5
Publications	7

## Discussion

This national survey found wide variability in fellowship interview practices, including inconsistent use of evidence-based interview techniques and frequent incorporation of multidisciplinary interviewers. Despite this variability, interview performance was highly valued in applicant ranking, even though fewer programs reported using formal interviewer training or standardized assessment tools. This finding highlights a disconnect between the recognized importance of interviews in applicant ranking and the inconsistent application of structured approaches to support reliable and equitable evaluation.

These findings are consistent with prior GME literature demonstrating gaps between recommended interview practices and their real-world implementation [5,6]. Structured interview components such as standardized questions, scoring rubrics, and interviewer training have been associated with reduced bias and improved reliability, yet adoption remains uneven across specialties [1-4]. Similar to studies in other fellowship settings, programs in this study placed greater weight on qualitative assessments, such as interview performance and letters of recommendation, than on traditional academic metrics such as publications [14-16]. This pattern reinforces the central role of interviews in holistic applicant assessment and underscores the potential impact of interview structure and interviewer preparation on selection outcomes.

The frequent inclusion of multidisciplinary interviewers reflects broader trends in interprofessional education and team-based clinical care. Although limited data exist regarding this practice in GME recruitment, prior work outside fellowship settings suggests that multidisciplinary interview panels may provide diverse perspectives and mitigate individual interviewer bias [8,9]. In CAP, where clinical care routinely involves collaboration with social workers, advanced practice providers, fellows, and other team members, the involvement of these professionals in fellowship interviews may align recruitment practices with the realities of clinical work. This approach may also signal program values related to collaboration, communication, and team functioning to applicants during the recruitment process. However, this hypothesis was not directly assessed in this study and warrants further investigation from the applicant's perspective.

However, the effectiveness of multidisciplinary interviewing in improving assessment quality, equity, or applicant experience remains uncertain. Variability in interviewer roles, training, and interview structure may influence how multidisciplinary input is integrated into selection decisions. Further work is needed to understand how interviewer composition interacts with structured interview practices and how these elements collectively influence applicant evaluation, program messaging, and downstream outcomes across GME.

This study has several limitations. First, data were self-reported and reflect PD perspectives, which may not fully capture actual interview practices or the experiences of other interview participants. Second, the response rate introduces the possibility of nonresponse bias, as programs with greater interest in interview practices may have been more likely to participate. Third, while the response rate was high, the absolute sample size was small, given the limited number of accredited CAP fellowship programs, which may limit statistical power and generalizability. Fourth, this study reflects the 2022-2023 interview season, during which all CAP fellowship interviews were conducted virtually; interview structure and interviewer interactions may differ from in-person formats. Finally, this descriptive study did not assess applicant experiences, match outcomes, or measures of bias reduction, limiting conclusions regarding the effectiveness of specific interview approaches.

## Conclusions

Fellowship interview practices vary widely, with inconsistent use of evidence-based interview structures despite the central role interviews play in trainee selection. In team-based subspecialties such as CAP, interviews function not only as assessment tools but also as a potential opportunity to reflect aspects of professional practice within the field. The inclusion of multidisciplinary interviewers by many programs suggests an emerging recruitment approach that aligns interviewer composition with interprofessional clinical models observed in practice. However, the impact of these approaches on applicant perceptions, equity, or selection outcomes was not assessed in this study. Future work is needed to evaluate how structured interview practices and multidisciplinary interviewer involvement influence applicant experience, assessment reliability, and downstream outcomes in GME.

## Appendices

Child Abuse Pediatrics Fellowship Interviews

By completing the survey below, you are consenting to participate in this research study.

Thank you!

What is your fellowship program's associated hospital?       _______________

How many faculty FTE are in your program? 

o   <2.1

o   2.1-5.0

o   5.1-8.0

o   8.1-11.0

o   11.1-14.0

o   >14.0

How many faculty members are involved with the interview process?

o   1-4

o   5-8

o   9-12

o   13-16

o   >16

Averaged over the last 3 years, how many applicants did your fellowship program interview each year? 

o   1-10

o   11-15

o   16-20

o   21-25

o   26-30

o   >30

Averaged over the last 3 years, how many fellowship positions were available each year? _______________

Averaged over the last 3 years, how many fellows were enrolled each year?    _______________

For the 2022-2023 interview season, did your fellowship program use interviewers other than your departmental faculty?

o   Yes

o   No

Who interviewed in addition to the departmental faculty?

o   Nurses

o   Advanced practice providers (APNs, PAs)

o   Social work

o   Behavioral health specialists (psychology)

o   Therapies (OT, PT, speech, athletic trainers, dietitians)

o   Physician faculty from other departments

o   Current fellows

o   Pharmacists

o   Administrative staff - please specify                _______________

o   Other - please specify               _______________

A panel interview is conducted by a group of two or more interviewers. For the 2022-2023 interview season, were non-physician interviewers included on a panel interview?

o   Yes

o   No, they have one-on-one interviews

Standardized or structured interview questions are pre-determined questions that are asked to all applicants. For the 2022-2023 interview season, did your program require the use of standardized or structured questions?

o   Yes

o   No

Blinded interviewers have limited knowledge regarding some or all of the candidate's application. For the 2022-2023 interview season, did your program use blinded interviewers?

o   Yes

o   No

What information did interviewers have?

o   MyERAS Application

o   Curriculum vitae

o   MSPE

o   Personal Statement

o   Photo

o   Letters of recommendation

o   Board transcript (USMLE or COMLEX-USA)

o   Medical school transcript

o   Other - please specify               _______________

For the 2022-2023 interview season, did your program use group interviews (more than one applicant being interviewed at a time)?

o   Yes

o   No

Multiple mini-interviews are an interview process similar to an objective structured clinical examination (OSCE) for assessing clinical performance. It typically includes 8-10 different stations, each lasting 5-10 minutes. For the 2022-2023 interview season, did your program use multiple mini-interviews?

o   Yes

o   No

For the 2022-2023 interview season, did your program use an established scoring rubric?

o   Yes

o   No

For the 2022-2023 interview season, were interviewers formally trained in interviewing? For example, did they complete faculty development sessions and/or implicit bias training and/or have a group review of scoring rubrics?

o   Yes

o   No

How often were they trained? By whom?     _______________

For the 2022-2023 interview season, how was your interview day structured?

o   Half day, (e.g., 8 AM to 12 PM)

o   Full day (e.g., 8 AM to 4 PM)

o   Other - please specify                _______________

For the 2022-2023 interview season, how many applicants were interviewed during one interview day?

o   1

o   2-4

o   >4

For the 2022-2023 interview season, how many separate interviews did each applicant undergo as part of the interview day?

o   1

o   2

o   3

o   4

o   5

o   >5

Please rate each of the following selection criteria for ranking applicants from 1 to 9 (1=most valuable; 9=least valuable).

o   USMLE/COMLEX-USA scores

o   Medical school rank

o   Letter from the director of applicant's residency program

o   Letter from a child abuse attending whom the applicant works with

o   Personal statement

o   Quality of residency program

o   Quality of interview

o   Publications

o   Applicant has expressed interest in the program (telephone call, email)
